# Fast and accurate Bayesian optimization with pre-trained transformers for constrained engineering problems

**DOI:** 10.1007/s00158-025-03987-z

**Published:** 2025-04-10

**Authors:** Rosen Ting-Ying Yu, Cyril Picard, Faez Ahmed

**Affiliations:** 1 Department of Mechanical Engineering, Massachusetts Institute of Technology, 77 Massachusetts Ave, Cambridge, MA 02139 USA; 2 Center for Computational Science and Engineering, Massachusetts Institute of Technology, 77 Massachusetts Ave, Cambridge, MA USA

**Keywords:** Bayesian optimization, Engineering design optimization, Machine learning, Surrogate-based optimization

## Abstract

Bayesian Optimization (BO) is a foundational strategy in engineering design optimization for efficiently handling black-box functions with many constraints and expensive evaluations. This paper introduces a novel constraint-handling framework for Bayesian Optimization (BO) using Prior-data Fitted Networks (PFNs), a foundation transformer model. Unlike traditional approaches requiring separate Gaussian Process (GP) models for each constraint, our framework leverages PFN’s transformer architecture to evaluate objectives and constraints simultaneously in a single forward pass using in-context learning. Through comprehensive benchmarking across 15 test problems spanning synthetic, structural, and engineering design challenges, we demonstrate an order of magnitude speedup while maintaining or improving solution quality compared to conventional GP-based methods with constrained expected improvement (CEI). Our approach particularly excels at engineering problems by rapidly finding feasible, optimal solutions. This benchmark framework for evaluating new BO algorithms in engineering design will be published at https://github.com/rosenyu304/BOEngineeringBenchmark.

## Introduction

Black-box optimization is a prevalent approach in engineering design optimization, particularly when dealing with problems where the objective function or constraints defining the set are unknown or ambiguous. This method is instrumental in navigating complex design spaces by optimizing solutions without a clear understanding of the underlying functions (Bajaj et al. 2021; Alarie et al. 2021; Tao et al. 2021). Lately, Bayesian optimization (BO) has emerged as a widely adopted black-box optimization tool for its ability to enable evaluations of functions with rapid speed. By leveraging probabilistic models and iteratively selecting points for evaluation, BO efficiently explores and exploits the design space, making it the forefront of active sampling for optimization (Eriksson and Poloczek 2021; Garnett 2023; Shahriari et al. 2015; Du et al. 2023).

Although BO has emerged as a promising tool for accelerating the search process, the full realization of its potential in engineering design is hindered by the constraint-handling ability of optimization algorithms (Greenhill et al. 2020; Cardoso et al. 2024). A common challenge in design involves identifying products that respect all constraints in the feasible design space. Particularly in structural design optimization, constraints such as cost limitations, regulatory requirements, material constraints, geometric considerations, manufacturing limitations, safety criteria, and ergonomic factors complicate the exploration of design landscapes (Gardner et al. 2017; Baptista and Poloczek 2018; Mathern et al. 2021). Therefore, there is an increase in attention on constraint-handling BO (CBO) algorithms in both engineering and computer science communities (Eriksson and Poloczek 2021; Biswas and Hoyle 2021; Kamrah et al. 2023; Gardner et al. 2014; Ragueneau et al. 2024; Tran et al. 2019, 2022; Ghoreishi and Allaire 2019).

BO’s algorithm limitations also come from its commonly used surrogate: the Gaussian Process (GP). By modeling a function with the mean and the kernel (covariance) function, GP suffers from cubic time complexity ${\mathcal {O}}(n^{3})$ of *n* training points, leading to scalability issues and runtime concerns (Liu et al. 2020; Gilboa et al. 2013). The need to repeatedly refit and infer in GP-based BO exacerbates the computational time demands. Moreover, the conventional approach for handling constraints in BO, the constrained expected improvement (CEI) method (Gelbart et al. 2014), requires a separate GP for each constraint, making the time scale with the number of constraints. The cubic time complexity, GP scalability issues, and need for multiple GPs to handle constraints make traditional BO methods particularly limiting when dealing with optimization problems with many constraints, as the computational burden grows substantially with increased dimensionality and number of constraints. Therefore, studies have focused on accelerating GP-based BO and improving GP’s scalability GP (Cunningham et al. 2008; Foreman-Mackey et al. 2017; Klein et al. 2017; Martinez-Cantin 2018; Pleiss et al. 2020).

To address the runtime limitations, Müller et al. (2023) have proposed a novel zero-training transformer framework known as Prior-data Fitted Networks for BO (PFNs4BO), which bypasses the fitting phase by leveraging a pre-trained model. The center of this framework is the Prior-data Fitted Network (PFN), a transformer architecture meta-trained on a vast dataset of synthetically generated priors. Unlike traditional hybrid approaches merging machine learning models with optimization techniques that require retraining for different problems, PFN is a problem-agnostic pre-trained model capable of solving diverse problems without additional training (Khan et al. 2020, 2022; Ali et al. 2020; Zhang et al. 2021). This pre-trained nature also enables PFN to produce posterior predictive distributions without additional training, offering a promising solution for BO by speeding up the optimization process by an order of magnitude. Although PFNs4BO has demonstrated a substantial speed improvement, its applicability has been confined to single-objective optimization problems without constraints.

Despite the computer science and engineering community’s numerous novel BO algorithms, there is a scarcity of open-source benchmark test suites, particularly for constrained engineering optimization problems. Various studies assess BO algorithms on distinct problem scenarios, such as material discovery (Liang et al. 2021) or chemical engineering experiments (Shields et al. 2021), making it challenging to compare methods across a broad range of engineering applications. Common optimization benchmark sets include COCO (Hansen et al. 2021), special sessions of competition in optimization at the Congress on Evolutionary Computation (IEEE CEC) and the Genetic and Evolutionary Computation Conference (GECCO), and Pymoo (Blank and Deb 2020). However, most of these benchmarks consist of synthetic numerical problems that may not accurately represent the challenges of engineering design problems (Picard and Schiffmann 2021). Of the published engineering design problems, few have their code publicly available while others are not easily interfaced with state-of-the-art BO libraries, limiting their accessibility and utility (Gandomi et al. 2011; Yang and Hossein Gandomi 2012; Eriksson and Poloczek 2021; Jetton et al. 2024). Additionally, most constrained engineering optimization problems in the literature are tested with Genetic Algorithms (GA; Gandomi et al. 2011; Yang and Hossein Gandomi 2012), making it difficult for researchers interested in testing with BO to find relevant studies. Overall, this results in a gap in the availability of a comprehensive engineering benchmark.

The focus of our study is to evaluate the performance of the recently published Prior-data Fitted Network (PFN)-based Bayesian Optimization (BO) in solving constrained engineering design optimization problems. We aim to demonstrate the effectiveness and potential superiority of general-purpose models that eliminate the need for fitting at each BO iteration, compared to traditional GP-based BO using common constraint-handling approaches. This could expand BO’s applicability to time-sensitive engineering tasks such as interactive experiment design assistance or robotics control optimization. Our contributions include:

1. *A pre-trained transformer-based CBO algorithm* we developed a constrained-handling PFN-based algorithm utilizing the Constrained Expected Improvement (CEI) acquisition function. Our PFN-CEI framework reduces the computational burden typically associated with GP-CEI by utilizing only one pre-trained surrogate model, rather than requiring multiple surrogates for objectives and constraints. The PFN model’s transformer architecture enables the simultaneous evaluation of objectives and constraints in a single forward pass. As a pre-trained model, PFN eliminates the need for iterative training, further enhancing its efficiency. PFN-CEI exhibits superior optimization performance compared to all other tested methods and is faster than GP-based CEI.
2. *Speed and performance comparison of CBO methods* we present three constraint-handling methods and two surrogate modeling approaches, evaluating their speed and optimization performance. We highlight that using PFN as the surrogate for CBO can achieve a **tenfold increase in speed compared to GP** and outperform GP with a superior anytime performance and feasibility rate. Additionally, we provide a reflective discussion on the potential of PFN-based BO as a fast optimization algorithm.
3. *Open-source code and enhanced benchmark tools* we present a set of 15 test problems for benchmarking BO algorithms, featuring engineering problems with many constraints from the literature. To foster collaborative progress, we make our constrained test problem set and corresponding Python codebase available at https://github.com/rosenyu304/BOEngineeringBenchmark, encouraging other researchers to build upon and advance engineering Bayesian optimization.

In this work, the background of engineering design optimization problems, Bayesian optimization with constraint-handling methods, and PFN’s application on BO are described in Sect. 2. Section 3 defines the constraint-handling BO algorithms of interest, the test problem set for benchmark, and the evaluation methods for CBO algorithms. The results of algorithm runtime and optimization performance are presented in Sect. 4. Finally, Sect. 5 discusses the overall performance of the tested CBO algorithms.

## Background

In this section, we introduce the common design optimization problems and methods, detail the Bayesian optimization algorithm, and highlight how the PFN-based BO method differs from traditional BO methods.

### Bayesian optimization for design optimization problems

Engineering design optimization problems are often formulated as one optimization objective subjected to many inequality constraints. The mathematical representation of such problems can be written in this form:

1 $$ \begin{gathered} \min _{x \in {\mathbb {R}}^{d}} \, f(x) \\ {\text {s.t}} \, g_{i}(x) \le 0, \quad i\in [1,G], \end{gathered} $$

where *x* is the design variable with dimension *d*, *f*(*x*) is the objective function, and $g_{i}(x)$ is the constraint with *G* as the numbers of constraints.

In general, finding the optimum of an optimization problem is non-trivial. The difficulty primarily stems from the ambiguity of objectives and constraints, coupled with the complexity of their evaluation. In engineering design, evaluating objective functions and constraints often involves physical experiments or complex simulations that are time-consuming and expensive. For instance, based on Ford Motor Company, conducting a car crash simulation may require 36 to 160 h per experiment (Wang and Shan 2006). Researchers must then update their dataset, run the optimization algorithm—a process that itself takes time—and repeat this cycle numerous times. This leads to slow data collection and resulting in a dataset too small for accurate predictions using machine learning-based surrogate models. Thus, BO, with its efficiency in data usage and ability to incorporate prior knowledge for surrogate-based global optimization, emerges as an ideal solution for engineering design optimization tasks.

BO is an active-learning algorithm designed for black-box optimization that iteratively improves performance through exploitation and exploration  (Garnett 2023). This process begins with a relatively small set of initial samples, typically ranging from 20 to 50 samples, depending on the complexity and dimensions of the problem at hand. BO employs a probabilistic surrogate model, commonly a Gaussian Process, to form a posterior belief about the design space. Utilizing this posterior, an acquisition function is then applied to determine the most promising next candidate, the one that is likely to be the optimum within the given space. Several acquisition functions commonly used in the literature are the probability of improvement (PI), expected improvement (EI), entropy search (ES), and upper confidence bound (UCB)  (Garnett 2015). Algorithm 1 demonstrates a general framework of the BO algorithm.

**Algorithm 1:**
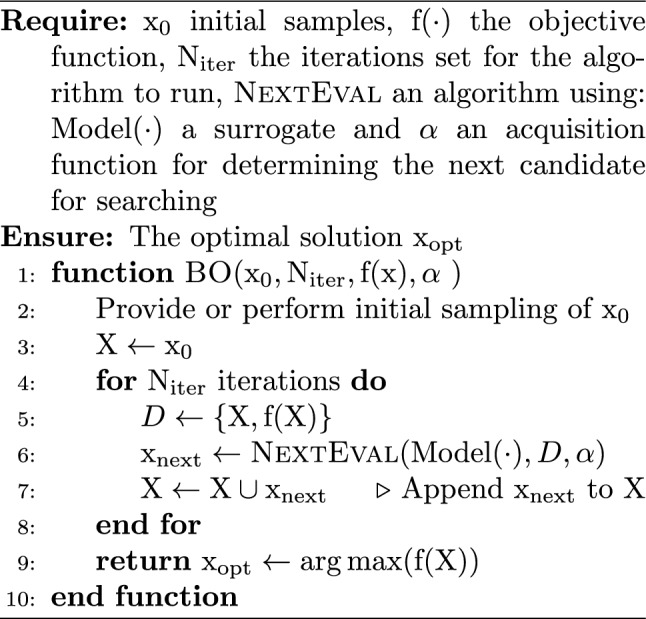
Bayesian optimization (BO)

### Constraint-handling Bayesian optimization (CBO)

Constraint-handling Bayesian optimization (CBO) has emerged as a key area in design optimization, addressing engineering limitations such as cost, ergonomics, safety, and regulatory standards. This study focuses on solving single-objective optimization problems with *G* constraints. Two main categories of constraint-handling approaches are typically employed for this type of problem: objective transformation and acquisition function modification.

#### Objective transformation

One common approach for constraint handling involves penalizing the objective value of infeasible data or increasing the objective value of feasible data through an objective function transformation. The penalty function (PF) is a widely-used method that alters the objective values of infeasible data (Fletcher 1975) by introducing a penalty term. Following this transformation, the BO algorithm is applied to the modified unconstrained optimization problem, aiming to minimize $f_{\text {PF}}$. In constrained optimization problems, the quadratic form of the penalty function is often employed, as illustrated in the following equation:

2 $$ f_{\text {PF}}(x) = f(x) + \rho \sum ^{G}_{i=1} \max (0, g_{i}(x))^{2}. $$

Equation (2) shows that, given one objective and *G* constraints to be optimized, $f_{\text {PF}}(x)$ is the penalty transformed objective that is calculated using the objective function *f*(*x*), the constraint functions $g_{i}(x), i \in [1,G]$, and $\rho$ is the penalty factor. The selection of the penalty factor value varies across different studies. In this paper, we initialize $\rho =1$ and multiply $\rho$ by 1.5 if the algorithm fails to identify an improved optimal value after five iterations (Jetton et al. 2024). However, a limitation of this method is its difficulty in implementation when the constraints cannot be represented analytically by numerical equations.

#### Acquisition function modification

As evaluating black-box constraints alongside the objective function through analytical transformation can be challenging, one strategy is to treat constraint functions as feasibility objectives. This requires surrogate modeling and their inclusion in the acquisition function calculation. Consequently, the constraint-handling Bayesian optimization (CBO) process employs $1+G$ surrogate models for modeling the objective function with *G* constraints. These surrogate models for constraints, known as feasibility models, determine the probability of feasibility $P_{\text {feas}}$ as shown in Eq. (3).

One of the most popular objective acquisition functions, expected improvement [EI, see Eq. (4)], has been adapted for constrained optimization.  Gelbart et al. (2014) proposed the constrained EI (CEI) acquisition function as the sum of EI and $P_{\text {feas}}$ for each constraint shown in Eq. (5).

3 $$ P_{\text {feas}} = \varPhi \left( \frac{- {\hat{g}}(x) }{\sigma _{g(x)}} \right) , $$

4 $$ \begin{aligned} \alpha _{\text {EI}} =&\left( f_{*} - {\hat{f}}(x)\right) \varPhi \left( \frac{f_{*} - {\hat{f}}(x) }{\sigma _{f(x)}} \right) \\&\quad + \sigma _{f(x)} {\mathcal {N}} \left( \frac{f_{*} - {\hat{f}}(x) }{\sigma _{f(x)}} \right) , \end{aligned} $$

5 $$ \alpha _{\text {CEI}} = \alpha _{\text {EI}} \prod _{i=1}^{G} P_{{\text {feas}}, {\text {i}}}, $$

where $\varPhi$ is the Gaussian cumulative distribution function (CDF), ${\hat{g}}(x)$ is the mean value of *g* at point *x*, $\sigma _{g(x)}$ is the standard deviation of *g*(*x*), $f_{*}$ is the minimum (optimum) observed value up until the current iteration, ${\hat{f}}(x)$ is the mean value of *f* at point *x*, $\sigma _{f(x)}$ is the standard deviation of *f*(*x*), and ${\mathcal {N}}$ is the Gaussian (Normal) distribution.

One limitation of CEI is that the combined probability of feasibility will be close to zero at the edge of the constraint regions. Therefore, a modified version of the CEI algorithm has been proposed to increase the chance of selecting solutions near the constraint boundaries (Bagheri et al. 2017):

6 $$ \alpha _{{\text {CEI}}+} = \alpha _{\text {EI}} \prod _{i=1}^{G} \min (1,2P_{{\text {feas}}, {\text {i}}} ). $$

### Prior-data fitted network and its application on Bayesian optimization

A Prior-Data Fitted Network (PFN) is a transformer framework trained to perform Bayesian inference (Müller et al. 2021). Just as large language models (LLMs) like ChatGPT learn to predict text from context (the prompt), PFNs learn to predict posterior distributions from observed data. The key advantage is its ability to generalize to new inputs without retraining—a limitation that has long challenged traditional Bayesian inference surrogate models that requires refitting for new datasets.

The PFN model undergoes a single meta-training phase, leveraging more than nine million synthetic datasets (Müller et al. 2021). These datasets integrate prior-posterior pairs from randomly initialized Gaussian Processes (GPs) with the well-engineered GP priors instantiated from Heteroscedastic Evolutionary Bayesian Optimization (HEBO; Cowen-Rivers et al. 2022). Through learning to predict the masked test data conditioned on training data, PFN learns to perform Bayesian inference. The one-time training on a diverse and large amount of datasets allows PFN to do in-context learning at inference time. The in-context learning ability enables PFN to process new query data ${\hat{x}}$ alongside the observed example dataset $D=${$(x_{1}, y_{1}), \ldots , (x_{k}, y_{k})$} in a single pass through its fixed-parameter model, similar to how humans draw from past experiences to tackle new but related tasks.

PFN model’s transformer architecture enables its in-context learning ability. The model first uses a linear layer to project both inputs *x* (feature data) and *y* (labels) from the dataset to a high-dimensional vector space (512 dimensions), encoding the dataset-level representation. Unlike LLMs where position matters for text understanding, here the data points are explicitly made position-invariant since the order of examples in a dataset shouldn’t matter for Bayesian inference. The encoded representation embeddings are then passed through the transformer encoder layers with the multihead attention mechanism. Within each attention layer, the model maps dataset example embeddings (encoded $x, \, y$ pairs) into queries, keys, and values. The attention mechanism then computes similarities between these representations, determining how strongly each input example and query point attends to the relevant data points in the dataset. Specifically, input examples can attend to other input examples to capture dataset-wide patterns, while query points can only attend to input examples to make predictions based on the observed data. During training, the model samples tasks from a prior distribution, draws data points and labels, masks one label, and learns to predict it based on the remaining points. The training process fixes a total number $N = n+m$ (where *n* is input size and *m* is queries) and samples *n* from a distribution to allow the model to handle different dataset sizes. At inference time, it takes a real dataset and test point as input and outputs a prediction distribution in a single forward pass (Müller et al. 2021).

With PFN’s ability to do Bayesian inference without retraining, PFN is integrated into the Bayesian optimization framework, named as PFNs4BO, as the surrogate to do efficient BO (Müller et al. 2023). By passing in the input observed data (*D*) and the query points (${\hat{x}}$) sampled from the unknown search space, PFN predicts the posterior distributions $p({\hat{y}}\, |\, {\hat{x}}, \, D)$ of the query points without retraining. With the PFN predicted posterior, acquisition values of the query points ($\alpha ({\hat{x}})$) can be calculated, and the sample with the highest acquisition value will be evaluated and appended to the observed data for the next search in the BO iterations. The key advantage of PFN-based BO over traditional GP-based approaches lies in its efficiency. While GP-based BO requires refitting at every iteration and relies on an acquisition function optimizer, PFN-based BO eliminates both requirements through its in-context learning ability.

The current released PFN model on the PFNs4BO GitHub repository^1^ is limited to 18 design variables and requires retraining for higher dimensions. While it supports three basic acquisition functions (EI, PI, and UCB), it can only perform unconstrained single-objective optimization. Our study expands this framework’s practical applications by introducing a constraint-handling acquisition function that leverages PFN’s transformer architecture for parallel data processing and optimization.

**Algorithm 2:**
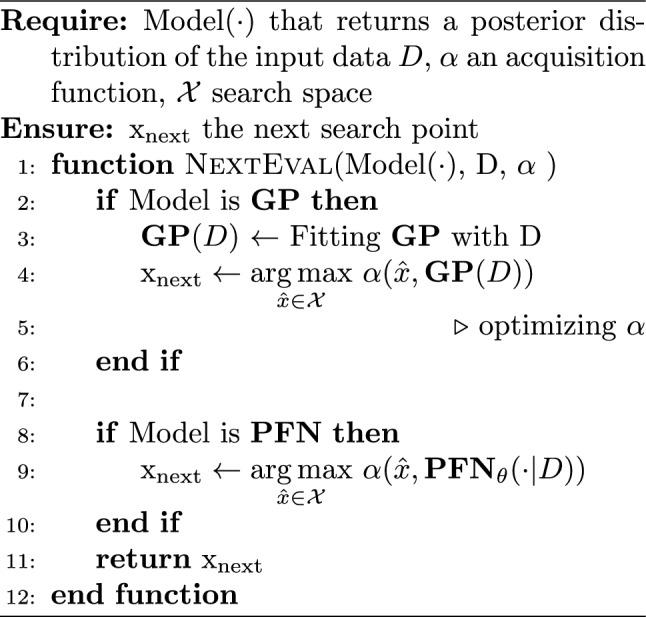
Surrogate modeling and acquisition function

## PFN-based CBO frameworks

**Fig. 1:**
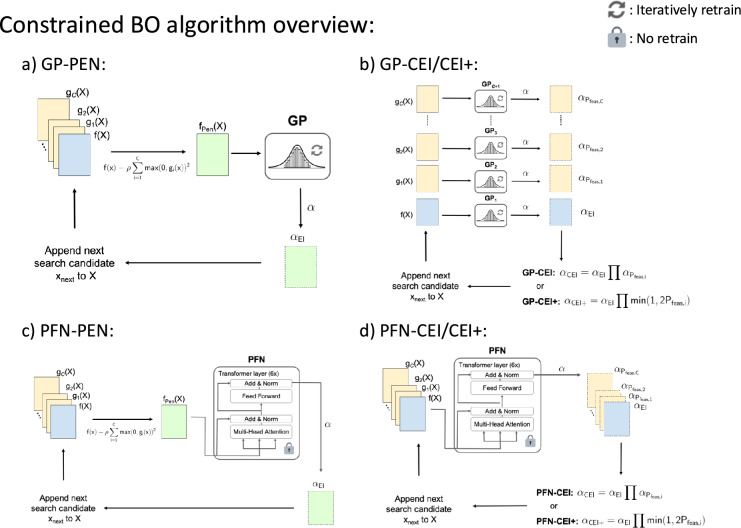
Comparison of constrained Bayesian optimization (BO) approaches: **a** GP-Pen: traditional GP-based BO with penalty function transformation, **b** GP-CEI/CEI+: given an objective and *G* constraints, GP-CEI requires $G + 1$ GPs to perform one search iteration for BO, with each GP needing to be fit and updated in every iteration, **c** PFN-Pen: PFN-based BO with penalty function transformation, and **d** PFN-CEI/CEI+: only one PFN is needed for optimizing an objective and *G* constraints, and no fitting of PFN occurs during BO since it is a pre-trained model. PFN’s transformer architecture allows the EI of the objective and $P_{\text {feas}}$ of the constraints to be solved in parallel in one pass without retrain

Here we propose three PFN-based CBO frameworks leveraging transformer architecture to handle constraints. While traditional GP-based methods require separate models for each constraint, our approach leverages the parallel processing capabilities of transformer architectures to handle multiple constraints efficiently. We present three different constraint-handling approaches: PFN-Pen (PFN with penalty function), PFN-CEI (PFN with constrained EI), and PFN-CEI+ (PFN with modified constrained EI). Figure 1 visualizes the difference between GP-based and PFN-based BO.

### PFN-Pen

Using the penalty transform method discussed in Sect. 2.2.1, PFN-Pen performs Bayesian optimization on the transformed objective $f_{\text {PF}}(X)$ and outputs the posterior for acquisition function $\alpha _{\text {EI}}$. For calculating $f_{\text {PF}}(X)$, we initialize the penalty factor $\rho =1$ and multiply $\rho$ by 1.5 when the algorithm fails to identify an improved optimal value after five iterations (Jetton et al. 2023). PFN’s pre-trained nature eliminates the need for repeated model fitting at each iteration when using GP.

7 $$ \alpha _{\text {EI}} ( {\mathbf {PFN}}_{\theta } (\cdot | \{X,f_{\text {PF}}(X)\} ). $$

### PFN-CEI

To implement CEI constraint-handling method stated in Sect. 2.2.2, the calculation of $\alpha _{\text {EI}}$ and $P_{\text {feas}}$ of each constraint function are required. In contrast to the GP-based approach, which requires a separate GP for each objective and constraint, a PFN can solve for the acquisition values for $\alpha _{\text {EI}}$ and $P_{\text {feas}}$ in one forward pass using a single surrogate. Leveraging the transformer architecture of PFN, which supports batch processing, we develop a method to simultaneously solve objectives and constraints with a single model. This architectural advantage significantly reduces computational overhead, especially for problems with many constraints. Figure 1’s b, d highlight the differences between GP-CEI and PFN-CEI.

8 $$ \alpha _{\text {CEI}} ( {\mathbf {PFN}}_{\theta } (\cdot | \{X,f(X)\} ) ). $$

### PFN-CEI+

This method adds the modified CEI+ threshold mentioned in Sect. 2.2.2 to the PFN-CEI algorithm to handle constraint boundaries.

9 $$ \alpha _{{\text {CEI}}+} ( {\mathbf {PFN}}_{\theta } (\cdot | \{X,f(X)\} ) ). $$

## Experiments

This section describes our constrained optimization problems, on which constrain-handling Bayesian Optimization algorithms are tested, and the evaluation metrics.

### CBO algorithms

In this study, we focus on benchmarking the performance of three proposed PFN-based CBO algorithms as highlighted in Sect. 3 with the current state-of-the-art GP-based BO using BoTorch library (Balandat et al. 2020), an open-source Bayesian optimization tool based on PyTorch. To make a fair comparison between algorithms using two different surrogates, we implement the same constraint-handling methods on GP and formulate three GP-based CBO algorithms: GP-Pen, GP-CEI, and GP-CEI+. A detailed visualization of all six CBO algorithms tested in this study is detailed in Fig. 1.

### Test problems

**Fig. 2:**
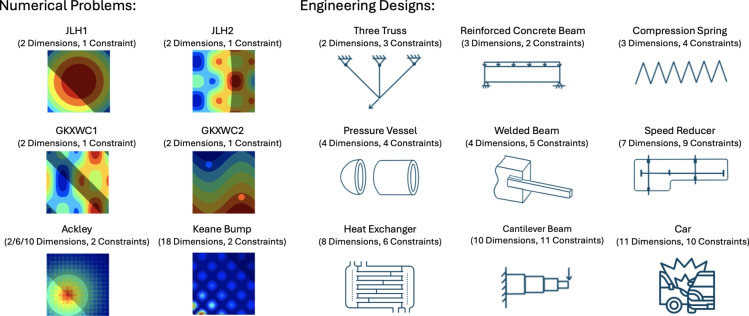
Overview of the details of the 15 benchmark problems. The non-feasible regions are shaded in the numerical problems. The Ackley problem is experimented with optimization in 2D, 6D, and 10D, resulting in 17 experiment problems

This study incorporates a diverse set of constrained test problems gathered from the literature of structural optimization algorithms  (Gandomi et al. 2011; Koziel and Yang 2011; Yang and Hossein Gandomi 2012; Jetton et al. 2023). With a focus on benchmarking the algorithm’s ability to solve engineering problems, we gather six numerical test problems and nine engineering design optimization problems with both continuous and discrete value optimization. These 15 problems are detailed in Fig. 2 and “Appendix”.

### Algorithm tests

The goal of the algorithm test is to provide a fair and comprehensive comparison of the state-of-the-art optimization methods. In this research, the optimization goal is to minimize the objective function for the test problems. The initial samplings for all test problems are performed with Latin Hypercube Sampling. Each test problem includes 50 sets of initial samples that are randomly selected, with each initial set representing a separate experimental trial. In each individual experimental trial, all six algorithms begin the optimization process with the same set of initial samples. Furthermore, each algorithm is run for 200 iterations of optimization and timed for the run time for each experimental trial. The achieved optimal value and the total CPU run time of the 50 optimization trials for each test problem are then evaluated using our ranking procedure.

All algorithms are run on the same computer and Python environment to ensure the speed comparison is fair. The CPU is an Intel$\circledR$ Core$^{\textrm{TM}}$ i9-13900K Processor with 24 cores, and 128 GB of RAM. The system is GNU/Linux 6.5.0-15-generic x86_{6}4 with Ubuntu 22.04.3 LTS as the operating system. While transformer models gain significantly from GPU acceleration and parallelization, we use only CPUs for a fairer comparison with CPU-run GPs.

### Evaluation metrics

#### Feasibility ratio

We define the optimization solution as the minimal value found by each method during optimization. However, for the BO algorithms utilized in this study, there is no guarantee of convergence to a feasible solution that respects all constraints. Therefore, the feasibility of the solution generated by each method is utilized as a metric for method evaluations. We define our constraint-handling performance evaluation methods as:

10 $${\text {Feasibility ratio}} = \frac{\#\,{\text {Trials with feasible solution}}}{{\text {Total}} \# {\text{trials}}=50}. $$

#### Statistical ranking

In this study, we employed statistical ranking methods rather than raw performance values to analyze the overall performance of the benchmarked BO algorithms. This ranking approach effectively normalizes the scale differences between problems, as some optimization tasks naturally yield larger objective values than others. Additionally, rankings help mitigate the impact of outliers and extreme values that could otherwise skew the comparative analysis.

For the statistical analysis, we utilized the Friedman test and Wilcoxon signed-rank test with Holm’s $\alpha$ correction. These statistical methods were chosen for their particular strengths in handling algorithm benchmarking data. As non-parametric tests, they make no assumptions about the underlying distribution of the performance metrics, which is essential since optimization results often exhibit non-normal distributions and contain outliers. The dependency structure in our experimental setup, where algorithms were tested using identical initial samples and seeds across the same set of experiment trails, is effectively addressed by these methods. The Wilcoxon signed-rank test is specifically designed for dependent paired comparisons, while the Friedman test accounts for the blocking effect of different test problems. Furthermore, when dealing with multiple algorithm comparisons, Holm’s $\alpha$ correction helps control the family-wise error rate (Ismail Fawaz et al. 2019; Brockhoff and Tušar 2023; Picard and Ahmed 2024).

#### Fixed-budget analysis

Hansen et al. (2022) presented the concept of fixed-budget evaluations, a technique for comparing the efficiency of optimization algorithms by allotting specific computational resources for their execution. Our investigation employs two distinct fixed-budget analysis methodologies:

1. *Fixed-iteration approach* the performance of each optimization algorithm is evaluated after a pre-determined number of 200 iterations.
2. *Fixed-runtime approach* the performance outcomes of the algorithms are compared within an identical CPU time frame. In this study, the runtime budget is set to be the time required for the fastest method to execute 200 iterations.

## Results

### Feasibility ratio performance

The feasibility ratio quantifies the capability of identifying a useful solution within the constrained space after a fixed number of iterations. Table 1 presents the feasibility ratio for each method across the test problems. For most test problems, all methods successfully find a feasible solution. Yet, not all algorithms can find a feasible solution for the Ackley function (2D, 6D, 10D), GKXWC2, and the Heat Exchanger problem every time. In these more challenging problems, we note that algorithms employing CEI for constraint handling exhibit a higher feasibility ratio than those utilizing a penalty function. For instance, in the Ackley 10D problem, GP-Pen achieves 32% feasible results, while GP-CEI and GP-CEI+ reach 86% and 78% feasibility rates, respectively. This trend is even more evident in the Heat Exchanger example. The feasibility ratio for GP-based methods increases from 2 to approximately 80% with the implementation of CEI, and for PFN-based methods, it rises from 40 to 100% when switching from the penalty transform to CEI.

The feasibility ratio analysis also reveals that the simplest method, GP-Pen exhibits the lowest feasibility rate, as expected. For problems with relatively higher dimensions, such as Ackley 10D and Heat Exchanger, PFN-based constrained BO methods demonstrate a higher feasibility rate than GP-based methods overall.

**Table 1:** Feasibility ratio comparison across different constraint-handling Bayesian optimization methods for 17 test cases. The feasibility ratio represents the percentage of trials (out of 50) where each method successfully found solutions respecting all constraints, thus, indicating algorithmic robustness. Higher percentages indicate better constraint-handling capabilities. The results highlight that PFN-based methods (particularly PFN-CEI and PFN-CEI+) consistently achieve superior feasibility rates, especially for challenging problems like Ackley 10D and Heat Exchanger, where traditional GP-based methods struggle

Test case	GP-Pen (%)	GP-CEI (%)	GP-CEI+ (%)	PFN-Pen (%)	PFN-CEI (%)	PFN-CEI+ (%)
JLH1	100	100	100	100	100	100
JLH2	100	100	100	100	100	100
GKXWC1	100	100	100	100	100	100
GKXWC2	92	100	100	100	100	100
Ackley 2D	98	100	100	100	100	100
Ackley 6D	100	98	98	100	100	100
Ackley 10D	32	86	78	92	94	92
Three Truss	100	100	100	100	100	100
Reinforced Concrete Beam	94	100	100	100	100	100
Compression Spring	100	100	100	100	100	100
Pressure Vessel	100	100	100	100	100	100
Welded Beam	100	100	100	100	100	100
Speed Reducer	100	100	100	100	100	100
Heat Exchanger	2	80	82	40	100	100
Cantilever Beam	100	100	100	100	100	100
Car	100	100	100	100	100	100
Keane Bump 18D	100	100	100	100	100	100

### Optimization performance at fixed iteration

**Fig. 3:**
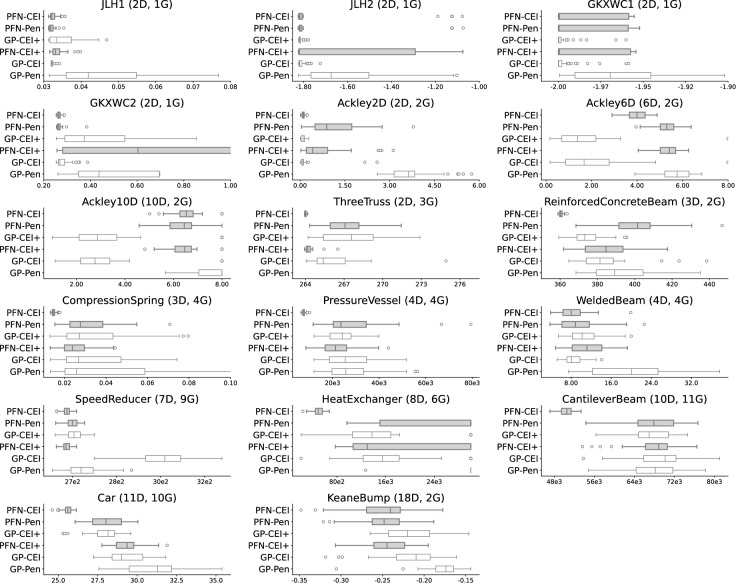
Box plots comparing the optimal value achieved by each optimization method across 17 benchmark problems. The methods are ordered vertically from best to worst overall performance according to the statistical ranking shown in Fig. 6, with PFN-CEI (the best performing method) at the top. Each box plot shows the distribution of optimal values found across 50 trials, where lower values indicate better optimization performance

Figure 3 displays the distribution of optimal and feasible solutions for each method across 17 problems of 200 iterations. Our analysis begins with the optimization performance of six CBO algorithms in different categories of optimization problems. For numerical problems such as Ackley 6D and 10D, GP-CEI+ is 60% and 68% better than PFN-CEI in optimization performance. However, note that these represent only the feasible samples, and Table 1 shows that GP-CEI+ only generates 78% feasible samples, while PFN-CEI generates 94% feasible samples for Ackley 10D. The Keane Bump 18D problem is known to be challenging for GP-based methods  (Eriksson and Poloczek 2021), where the PFN-based methods surpass the GP-based methods by 10%.

For the nine engineering problems, PFN-based methods consistently rank highest compared to GP-based methods. The median solutions from the PFN-CEI method dominate all engineering problems, exhibiting performance two to three times better than that of GP-CEI or GP-CEI+.

### Optimization performance at fixed-runtime

**Fig. 4:**
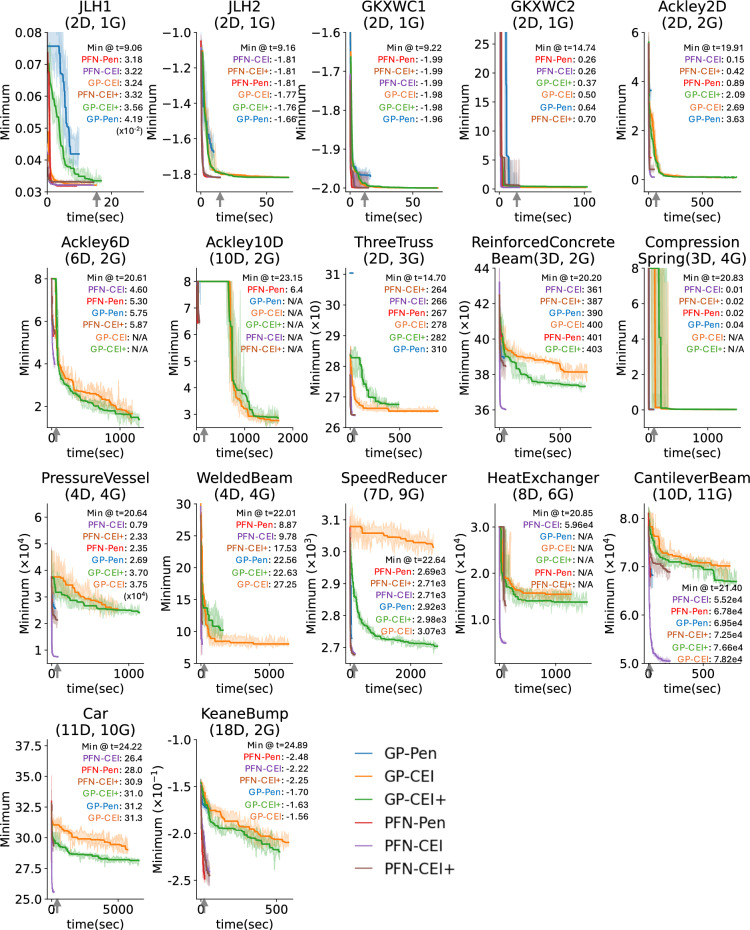
Convergence plots comparing performance of optimization methods across all benchmark problems. Each line shows the median optimization trajectory over 50 trials, with shaded regions indicating 95% confidence intervals. The plots track the 200-iteration optimization progress and each algorithm’s runtime. For fixed-runtime analysis, the runtime limit is defined by the completion time of PFN-Pen (the fastest method) reaching 200 iterations, where the gray arrow is pointing to

Figure 4 illustrates the convergence plot for each problem, highlighting the optimal value at a fixed time constraint marked by the completion of 200 iterations by PFN-Pen, the fastest approach. Upon the completion of PFN-Pen, a comparative analysis of performance outcomes reveals that PFN-CEI outperforms the others in 10 of the problems, while PFN-Pen leads in 6 cases, and PFN-CEI+ prevails in 1 case. PFN-based strategies consistently exhibit superior anytime performance throughout the operational timeframe defined by the termination of PFN-Pen.

Additionally, the convergence plot shows the advantage of PFN-based BO in limited runtime search, where GP-based BO sometimes cannot find feasible solutions. Specifically, for the Ackley 6D and 10D problems, although GP-based CEI methods outperform PFN-based methods after 200 iterations, the GP-based method is unable to find any constrained optimal solution within the given runtime limit. Even more evident, in the Compression Spring and Heat Exchanger problems, the PFN-based method outperforms in optimization, while GP-CEIs fail to find a feasible solution in the given time budget and perform worse than PFN-CEI at the fixed iteration.

### Speed performance

**Fig. 5:**
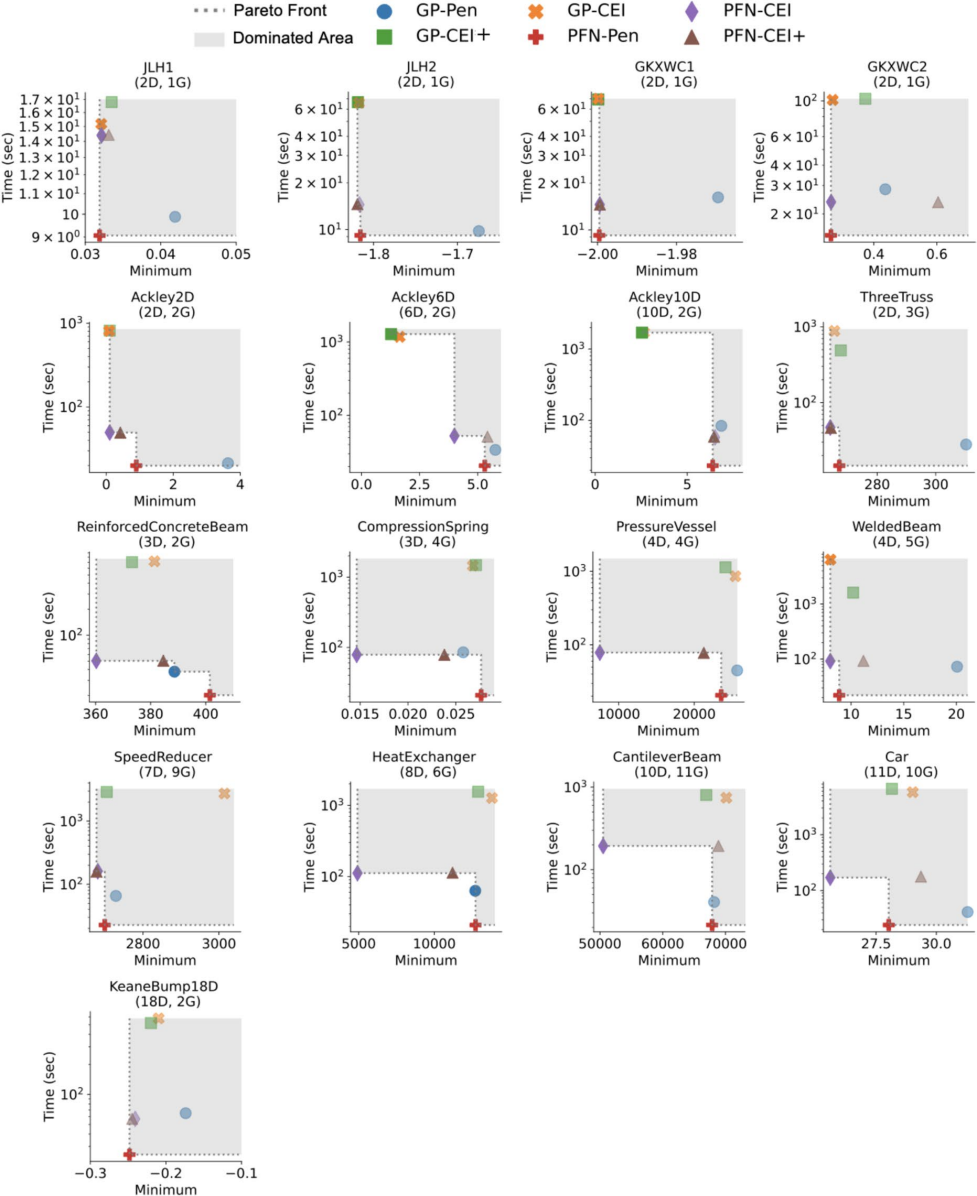
Pareto plots demonstrating the trade-off between performance and total execution time (log-scale) for each method and test problem. *D* is the objective dimension, and *G* is the number of constraints. The average Pareto rank of each method over 17 experiment trials is [GP-Pen, GP-CEI, GP-CEI+, PFN-Pen, PFN-CEI, PFN-CEI+] = [2.118, 2.353, 2.353, 1, 1.353, 1.765], where the smaller rank, the better, and rank 1 is the best. In problems with more than one constraint, PFN-based methods are 10 times faster than the GP-based CEI methods

In addition to the convergence plot in Fig. 4, Fig. 5 illustrates the Pareto trade-off between time and performance for each test problem. In both figures, algorithms using the penalty function are always faster than those utilizing CEI. PFN-Pen leads in speed on the Pareto front in all 17 benchmark problems, completing 200 iterations in 17.8 s on average. On the other hand, GP-Pen requires 36.5 s since GP is affected by the problems’ expanding dimensions.

The speed disadvantage of GP’s approach becomes more evident when comparing CEI methods for constraint handling. GP-CEI and GP-CEI+ need 647.9 and 590.8 s on average to perform 200 iterations of search, with a maximum of 6588.9 s (1.77 h) for running the Heat Exchanger problem. In contrast, PFN-CEI and PFN-CEI+ only need 55.8 s on average to finish an experiment, showing that they are 10 times faster than the GP-based CEI methods.

Moreover, CEI algorithm speeds drop as *G* goes from 1 to 11, with GP-based methods taking 430 times longer because of the feasibility calculations for each constraint. Due to the PFN’s capability to solve both objectives and constraints in a single forward pass, PFN-based CEI methods’ speeds only drop by a factor of 13, demonstrating the dominance of PFN-based methods in speed.

### Overall rank

**Fig. 6:**
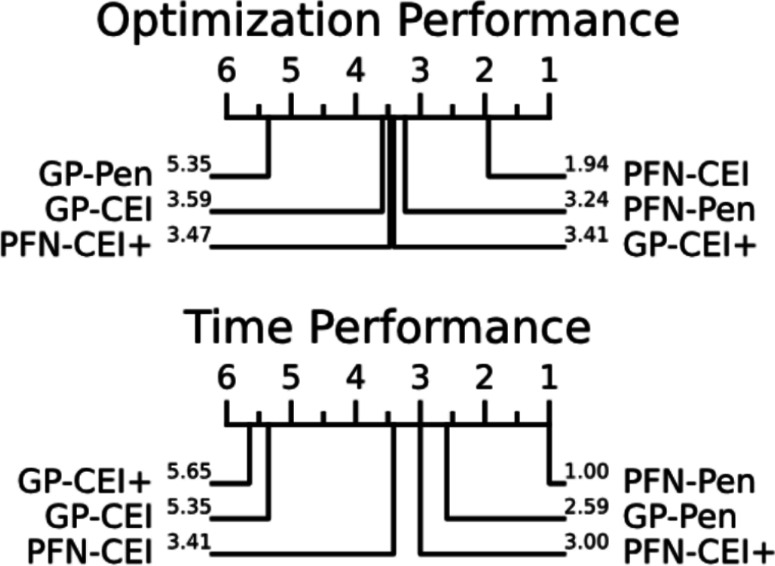
Critical difference rank plot of overall results. A smaller rank indicates a better result. Regarding optimization performance, two PFN-based methods (PFN-CEI and PFN-Pen) lead. For time performance, PFN-Pen dominates as the fastest method, while the GP-based CEI ranks last

Figure 6 shows the critical difference plot from the statistical ranking of the six different CBO approaches. As stated in Sect. 4.4.2, a smaller rank (towards the left) indicates a better performance. The optimization performance critical difference plot result shows that left-most two PFN-based methods, PFN-CEI and PFN-Pen, outperform the traditional GP-based methods for the optimization performance rank. Surprisingly, applying CEI+ onto PFNs does not help improve the performance as it does for GP-CEI+. Furthermore, the time performance critical difference plot results show that the two methods using the penalty transform require the least time, with the PFN-Pen being the fastest method. The GP-CEI and GP-CEI+ methods are the slowest as expected.

## Discussion

### Recommendations for constrained Bayesian optimization methods

Overall, the results show that using PFN as BO’s surrogate outperforms GP in speed and optimization performance. Notably, PFN algorithms, which are already 10 times faster than GP, could achieve even greater speeds through parallelization, as we tested all our models only on CPU. Considering the optimization performance, PFN-CEI achieves the highest ranking in optimization performance, followed by PFN-Pen. Although PFN-Pen completes 200 iterations more quickly, PFN-CEI achieves faster convergence, as shown in Fig. 4.

While PFN-based methods demonstrate both superior speed and optimization performance for most engineering problems, GP-based methods may still be preferred in specific scenarios. For numerical problems with complex landscapes like Ackley 6D and 10D, GP-CEI+ achieves better optimization performance despite being slower. Additionally, GP-based methods remain valuable when the problem dimension exceeds PFN’s current 18D limitation and when the problem characteristics significantly deviate from PFN’s training distribution.

Therefore, we recommend using PFN-CEI for overall performance and could do an early stop before 200 iterations if the user wants to perform optimization with a limited runtime budget. For specialized cases requiring high accuracy in numerical optimization or problem dimensions beyond 18D, GP-based methods remain a robust choice despite the speed trade-off.

### Potentials of pre-trained transformers-based BO

From the convergence plot and fixed-runtime analysis, it is evident that pre-trained-model-based BO is effective in rapidly assessing optimization problems and providing feasible solutions when GPs are unable to do so. The transformer architecture’s impact on BO performance manifests through three key mechanisms. First, its attention mechanism enables parallel processing of multiple constraints and objectives, allowing efficient handling of complex optimization problems. Second, the architecture’s ability to handle variable-sized inputs without retraining provides flexibility across different problem scales. Third, the efficient single-pass inference eliminates the need for iterative retraining, substantially reducing computational overhead.

Observing PFN’s capability for rapid optimization through the transformer’s ability to solve multiple functions in parallel in a single forward pass, we want to emphasize the potential of using pre-trained models for BO and its applications. Experimental optimization or user-guided BO (Jetton et al. 2024), which requires human input to the engineering optimization framework, will be time-sensitive as users must wait for BO to indicate the next potential optimum. The transformer-based approach’s rapid processing capabilities are particularly valuable in these interactive scenarios, where minimizing latency is crucial for maintaining effective user engagement. Fast BO enables users to receive immediate feedback, enhancing the efficiency of the workflow.

Conversely, BO for hyperparameter tuning has been shown to take an extended period to identify the best hyperparameters for large models in image classification or language modeling (Cho et al. 2020). The parallel processing capabilities and efficient inference characteristics of transformer-based BO could significantly accelerate this process, potentially revolutionizing the field of automated machine learning optimization. Transformer-based BO could potentially unlock the possibilities of fast hyperparameter optimization.

### Understanding the complexity of test problems

One metric for comprehending the intricacy of the constrained test problems is to evaluate the feasible ratio of the six methods for each problem. Multi-modal numerical problems like Ackley, and problems with relatively small constrained areas, such as GKXWC2, are particularly challenging since not all methods have a 100% feasible ratio. With higher feasibility ratios in most engineering test cases, BO proves effective for constrained engineering design problem-solving. The Heat Exchanger problem, however, demonstrates the lowest overall feasibility among all problems with the longest runtime due to the exclusive presence of independent variables in the constraints and not in the objective function, making it the most complex engineering problem.

An alternative metric for evaluating problems is the variance in results across methods, as shown in Fig. 3. The choice of BO method is particularly vital for numerical problems, where results vary greatly, especially in cases like JHL2, GKXWC1, GKXWC2, and Ackley, due to their complexity. Engineering problems such as Pressure Vessel and Speed Reducer also have large variances in their results, underscoring the importance of method selection.

### Limitations

This study provides insights into the application of PFN as black-box surrogates for BO while also acknowledging several inherent limitations.

#### Absence of acquisition function optimization

Firstly, the PFN-based methods employed in this research do not utilize acquisition function optimizers that are commonly implemented in BO algorithms. Theoretically, the absence of an acquisition optimizer could potentially accelerate the algorithm but suppress the optimization performance. While PFN-based BO still had the overall best performance, adding acquisition functions to them could further enhance their performance in multi-modal problems such as Ackley. Therefore, further evaluations of PFN-based BO utilizing standard acquisition optimization approaches are required.

#### Dimensionality and model scaling constraints

A key limitation of the current PFN-based methods is their restricted dimensionality capacity. However, the model can only process up to 20 design features (# of objective + constraints), constraining its applicability to real-world engineering problems that often have higher dimensionalities. While transformer models like PFN can be scaled up to handle more dimensions, this requires substantial computational resources, taking approximately 24 h of training even with eight RTX 2080 Ti GPUs (Müller et al. 2023). Additionally, transformers are memory-intensive with quadratic dependency on input length, limiting processing to around 5000 training points on current consumer GPUs. Future research should explore efficient architectures that can handle higher-dimensional engineering design spaces while maintaining computational feasibility.

#### Limited validation in real engineering applications

Additionally, while PFNs show promise in addressing our nine engineering design problems through BO, this limited scope may not capture the full complexity and diversity encountered in practical engineering situations. We hope that our research will encourage other scholars to adopt PFN-based BO for their engineering design tasks and to evaluate novel algorithms using our benchmark problem sets. We also aim to expand the benchmark problem based on community input, providing a standardized test bed for research in constrained BO methods.

#### Constraints in performance evaluation framework

Lastly, our evaluation metrics were limited to runtime speed and optimization performance in fixed iterations. Future studies should explore additional aspects of Bayesian optimization, such as the convergence rate of iterations and scalability.

### Future work

Our work in this paper lays the groundwork for several promising paths for future research. Recognizing that most engineering design problems involve multiple objectives, we aim to expand our constrained PFN-based BO methods to handle multi-objective optimization or active sampling by introducing additional acquisition functions or modifying the PFN architecture. Coupled with the dramatic speed improvements achieved by PFN-based methods, this expansion opens up opportunities for novel applications in time-sensitive domains. These include user-guided interactive BO for real-time design exploration, adaptive experiment design where quick decisions are crucial, and robotics control optimization requiring rapid adaptations. Additionally, PFN’s efficiency could enable real-time hyperparameter tuning for large-scale machine learning models, addressing a significant bottleneck in model development.

Furthermore, we plan to benchmark our approach against more constraint BO methods such as Scalable Constrained Bayesian Optimization (SCBO;  Eriksson and Poloczek 2021), as our current comparison is limited to CEI. This will involve a detailed comparison of PFN with other CBO methods using active sampling strategies or BO methods that employ neural networks instead of Gaussian Processes.

Another promising research direction involves tackling the challenge of high-dimensional optimization in Bayesian Optimization (BO). We aim to investigate the pre-training of larger transformer architectures tailored for higher-dimensional problems. Leveraging the rapid compute speed of PFNs, we also envision addressing high-dimensionality through strategies such as bootstrapping and aggregation, which hold significant potential for enhancing the performance of PFN models in this context.

Finally, we will continue expanding our benchmark suite based on community input, providing a standardized platform for evaluating new BO algorithms in engineering design. This expansion not only enhances the versatility of our approach but also opens up new possibilities for tackling complex optimization challenges in multidisciplinary optimization.

## Conclusions

This research evaluates a novel approach for constraint-handling Bayesian optimization (CBO) by utilizing prior-data fitted networks (PFN) to remove the need for refitting the Gaussian Process (GP) for every searching iteration. Our comprehensive analysis is supported by benchmarking the methods on the 17 constrained optimization experiments, ranging from numerical synthesized test cases to engineering design problems. By using three constraint-handling approaches, penalty function PF, constrained expected improvement CEI, and modified constrained expected improvement CEI+, and two different surrogates, we evaluate six different CBO algorithms: GP-Pen, GP-CEI, GP-CEI+, PFN-Pen, PFN-CEI, PFN-CEI. The results show that across the optimization problems, the PFN-based approach has dominated both performance and speed. PFN-CEI has the best optimization performance, followed by PFN-Pen and GP-CEI+, with exceptional performance in engineering problems. With the unique transformer architecture and pre-trained nature, PFN-based BO shows its capability to accelerate the BO process by an order of magnitude compared to GP-based Bayesian optimization.

## Appendix: Benchmark problems

### JLH1

This numerical problem is a two-dimensional “sphere” problem featuring a single optimum and a continuous inequality constraint as detailed in (Jetton et al. 2023). The domain of interest for both $x_{1}$ and $x_{2}$ is [0, 1].

$$\begin{aligned} &  f(x) = x_{1}^{2} + x_{2}^{2}, \\ &  g(x) = x_{1} + x_{2} + 0.5 \le 0. \end{aligned}$$

### JLH2

This numerical problem features a is two-dimensional objective with local optimum used in multiple research (Jetton et al. 2024; Gardner et al. 2014). The continuous inequality constraint is proposed by Jetton et al. (2024). The domain of interest is $x_{1}\in [-5,0]$ and $x_{2}\in [-5,5]$.

$$\begin{aligned} &  f(x) = \cos (2x_{1})\cos (x_{2}) + \sin (x_{1}), \\ &  g(x) = \frac{1}{4} (x_{1}+5)^{2} + \frac{1}{100} x_{2}^{2} - 1 \le 0. \end{aligned}$$

### GKXWC1

The objective function of this question is identical to JHL2. However, the unique discontinuous inequality constraint is created by Gardner et al. (2014). The domain of interest for both $x_{1}$ and $x_{2}$ is [0, 6].

$$\begin{aligned} &  f(x) = \cos (2x_{1})\cos (x_{2}) + \sin (x_{1}), \\ &  g(x) = \cos (x_{1})\cos (x_{2}) - \sin (x_{1})\sin (x_{2}) - 0.5 \le 0. \end{aligned}$$

### GKXWC2

This numerical problem is two-dimensional, featuring a multiple optimum and a discontinuous inequality constraint with a tiny feasible area proposed by Gardner et al. (2014). The domain of interest for both $x_{1}$ and $x_{2}$ is [0, 6].

$$\begin{aligned} &  f(x) = \sin (x_{1}) + x_{2}, \\ &  g(x) = \sin (x_{1})\sin (x_{2}) + 0.95 \le 0. \end{aligned}$$

### Ackley function

Ackley function is a popular scalable numerical test case for optimization. It has many local minimums with the global optimal at $x=[0,0,\ldots ]^{n}$ for a *n*-dimensional problem. We consider the domain of interest to be $[-5,10]^{n}$ with $n=2,6,10$. The constraints formulation is taken from Eriksson and Poloczek (2021). The large number of local minimum, multi-modal nature, and small feasible region makes this problem challenging.

$$\begin{aligned} f(x) &= -20\exp { \left( -0.2\sqrt{ \frac{1}{n} \sum ^{n}_{i=1} x_{i}^{2}} \right) } - \exp { \left( \frac{1}{n} \sum ^{n}_{i=1} \cos (2\pi x_{i}) \right) }  \\  &  \quad +20+\exp \left( 1 \right) , \\ g_{1}(x) &= \sum ^{n}_{i=1} x_{i} \le 0, \\ g_{2}(x)& = \Vert x \Vert _{2} - 5 \le 0. \end{aligned}$$

### Three truss

The objective of this problem is to minimize the volume of the three-bar truss while each truss is constrained by the stress acting on it. the given information are length (*L*) is 100 cm, pressure (*P*) is 2 kN/${\text {cm}}^{2}$, and stress ($\sigma$) is 2 kN/${\text {cm}}^{2}$. The domain of interest for both $x_{1}$ and $x_{2}$ is [0, 1]. The formulation is taken and varied from Yang and Hossein Gandomi (2012).

$$\begin{aligned} &  f(x) = ( 2\sqrt{2}x_{1} + x_{2} ) L, \\ &  g_{1}(x) = \frac{ (\sqrt{2}x_{1} + x_{2})P}{\sqrt{2}x_{1}^{2} + 2x_{1}x_{2}} - \sigma , \\ &  g_{2}(x) = \frac{x_{2} P}{\sqrt{2}x_{1}^{2} + 2x_{1}x_{2}} - \sigma , \\ &  g_{3}(x) = \frac{P}{ x_{1} + \sqrt{2}x_{2}} - \sigma . \end{aligned}$$

### Reinforced concrete beam

This 3D problem describes a beam supported at two end points spaced by 30 ft. subjected to a live load and a dead load. The optimization objective is to minimize the cost while satisfying the constraints imposed by the loads and the safety requirement from ACI 318-77 code. This problem is representative of discrete value optimization. The cross-sectional area of the reinforcing bar ($A_{\text {s}}$) and the width of the concrete beam (*b*) are discrete while the depth of the concrete beam (*h*) is continuous. The domain of interest for each objective variable is: $A_{\text {s}} \in [0.2, 15]$ and $h\in [5,10]$. As for *b*, it is the integers in [28, 40]. The formulation below is taken from Gandomi et al. (2011).

$$\begin{aligned} &  f(x) = 29.4A_{\text {s}} + 0.6bh, \\ &  g_{1}(x) = \frac{h}{b} - 4 \le 0, \\ &  g_{2}(x) = 180 + \frac{7.35A_{\text {s}}^{2}}{b} - A_{\text {s}}h \le 0. \end{aligned}$$

### Compression spring

This optimal spring design problem has many variations in the literature. Here we pick the helical compression spring design optimization problem proposed by Gandomi et al. (2011). The goal is to minimize the spring volume. The optimization variables are: the number of spring coils (*N*), the winding coil diameter (*D*), and the wire diameter (*d*). The domain of interest for each objective variable is $d\in [0.05, 1]$, $D\in [0.25, 1.3]$, and $N\in [1.5, 2]$.

$$\begin{aligned} &  f(x) = (N+2)Dd^{2}, \\ &  g_{1}(x) = 1 - \frac{D^{3}N}{71,785 d^{4} } \le 0, \\ &  g_{2}(x) = \frac{4D^{2}-Dd}{12,566(Dd^{3}-d^{4})} + \frac{1}{5108d^{2}} -1\le 0, \\ &  g_{3}(x) = 1 - \frac{140.45d}{D^{2}N} \le 0, \\ &  g_{4}(x) = \frac{D+d}{1.5} - 1\le 0. \end{aligned}$$

### Pressure vessel

The optimization of a cylindrical pressure vessel with both ends capped is to minimize the cost while meeting the ASME constraints on boilers and pressure vessels. The objective (cost) is defined with four optimization variables [thickness of the cylindrical skin ($T_{\text {s}}$), thickness of the spherical head ($T_{\text {h}}$), the inner radius (*R*), and length of the cylindrical segment of the vessel (*L*)]. The domain of interest for both $T_{\text {s}}$ and $T_{\text {h}}$ is 0.0625*T*, where *T* is a random integer value from 1 to 99. For both *R* and *L*, the domain of interest is [10, 200]. The problem is originally proposed by Sandgren (1990) and the formulation is taken and varied from Gandomi et al. (2011).

$$\begin{aligned} f(x)&= 0.6224T_{\text {s}} R L + 1.7781T_{\text {h}} R R \\  &  \quad + 3.1661T_{\text {s}} T_{\text {s}} L + 19.84T_{\text {s}}T_{\text {s}} R, \\ g_{1}(x) &= -T_{\text {s}} + 0.0193R \le 0, \\ g_{2}(x)& = -T_{\text {h}} + 0.00954R \le 0, \\ g_{3}(x) &= \pi R^{2}L - 4/3 \pi R^{3} + 1,296,000 \le 0, \\ g_{4}(x) &= L-240 \le 0. \end{aligned}$$

### Welded beam

The welded beam is designed to minimize the manufacturing cost. The five constraints are imposed on the shear stress, bending stress in the beam, geometry, buckling load on the beam, and deflection of the beam. The optimization variables are the thickness of the weld (*h*), the length of the welded joint (*l*), the width of the beam (*t*), and the thickness of the beam (*b*). The domain of interest is $h\in [0.125,10], l\in [0.1,15], t\in [0.1,10]$, and $b\in [0.1,10]$. Details about the problem formulation can be found in Gandomi et al. (2011).

$$\begin{aligned}   f(x) = 1.10471h^{2}l + 0.04811tb(14+l), \\   \tau (x) = \sqrt{ \frac{ \tau '(x) ^{2} + \tau ''(x)^{2} + l\tau '(x)\tau ''(x)}{ \sqrt{0.25(l^{2} + (h+t)^{2})} } }, \\   \tau '(x) = \frac{ 6000 }{ \sqrt{2}hl }, \\   \tau ''(x) = \frac{ 6000(14+0.5l) \sqrt{0.25(l^{2} + (h+t)^{2} )} }{ 2 (0.707hl \left( \frac{l^{2}}{12} + 0.25(h+t)^{2} ) \right) }, \\   \sigma (x) = \frac{ 504,000 }{ t^{2} b }, \\   P_{\text {c}}(x) = 64,746(1-0.0282346t)tb^{3}, \\ \delta (x) = \frac{ 2.1952 }{ t^{3} b }, \\   g_{1}(x) = \tau (x) - 13,600,   \le0\\   g_{2}(x) = \sigma (x) - 30,000,  \le 0\\   g_{3}(x) = b-h,   \le0\\  g_{4}(x) = P_{\text {c}} - 6000,  \le 0\\   g_{5}(x) = 0.25 - \delta (x). \end{aligned}$$

### Speed reducer

The speed reducer design is one of the most famous benchmark problems in structural engineering. The objective is to minimize the weight of the speed reducer while considering the dimensions of the inner bearings and shafts. The domain of interest is $b\in [2.6,3.6], m\in [0.7,0.8], z\in [17,28], L_{1}$ and $L_{2}\in [7.3, 8.3], d_{1}\in [2.9,3.9],$ and $d_{2}\in [5,5.5]$. The problem originated from Golinski (1973) and the formulation is taken from Yang and Hossein Gandomi (2012).

$$\begin{aligned} f(x) &= 0.7854bm^{2} (3.3333z^{2} + 14.9334z \\  &  \quad - 43.0934) - 1.508b(d_{1}^{2} + d_{2}^{2}) + 7.4777(d_{1}^{3} + d_{2}^{3}) \\  &  \quad + 0.7854(L_{1}d_{1}^{2} + L_{2}d_{2}^{2}) ), \\ g_{1}(x) &= \frac{27}{bm^{2}z} - 1 \le 0, \\ g_{2}(x) & = \frac{397.5}{bm^{2}z^{2}} -1 \le 0, \\ g_{3}(x) &= \frac{1.93L_{1}^{3}}{mzd_{1}^{4}} -1 \le 0, \\ g_{4}(x) &= \frac{1.93L_{2}^{3}}{mzd_{2}^{4}} -1 \le 0, \\ g_{5}(x) &= \frac{ \sqrt{ \left( \frac{745L_{1}}{mz} \right) ^{2} + 1.69\times 10^{6} } }{110d_{1}^{3}} -1 \le 0, \\ g_{6}(x) &= \frac{ \sqrt{ \left( \frac{745L_{2}}{mz} \right) ^{2} + 157.5\times 10^{6} } }{85d_{2}^{3}} -1 \le 0, \\ g_{7}(x) & = \frac{mz}{40} -1 \le 0, \\ g_{8}(x) &= \frac{5m}{b-1} -1 \le 0, \\ g_{9}(x) &= \frac{b}{12m} -1 \le 0. \end{aligned}$$

### Heat exchanger design

The Heat Exchanger design is known for its six complicated binding constraints. Although the objective is simple, satisfying all linear ($g_{1} \sim g_{3}$) and nonlinear constraints ($g_{4} \sim g_{6}$) make it a difficult constrained optimization problem. The domain of interest is $x_{1}\in [10^{2}, 10^{4}]$, $x_{2\sim 3}\in [10^{3}, 10^{4}]$, and $x_{4\sim 8}\in [10, 10^{3}]$. Details of the problem formulation can be found here (Yang and Hossein Gandomi 2012).

$$\begin{aligned} f(x) &= x_{1}+x_{2}+x_{3}, \\ g_{1}(x) &= 0.0025(x_{4}+x_{6}) - 1 \le 0, \\ g_{2}(x) &= 0.0025(x_{5} + x_{7} - x_{4}) - 1 \le 0, \\ g_{3}(x) &\; <=0\;= 0.01(x_{8}-x_{5}) - 1, \le 0,\\ g_{4}(x) &= 833.33252x_{4} + 100x_{1} - x_{1}x_{6} \\  &  \quad - 83,333.333\le 0, \\ g_{5}(x) &= 1250x_{5} + x_{2}x_{4} - x_{2}x_{7} - 125x_{4} \le 0, \\ g_{6}(x) &= x_{3}x_{5} - 2500x_{5} - x_{3}x_{8} + 1,250,000 \le 0. \end{aligned}$$

### Cantilever stepped beam

This problem is originally proposed to minimize the volume of the stepped cantilever beam by Thanedar and Vanderplaats (1995). In this 10D problem, we optimize a five-stepped cantilever beam with variable width $x_{i}$ and height $x_{i+5}$ of each step. The constant used are total length $L=100$, Young’s modulus $E = 2 \times 107\,{\text {GPa}}$, and force applied on the end of the beam $P = 50,000\,{\text {N}}$. The domain of interest for $x_{1\sim 5}$ is [1, 5] and for $x_{6\sim 10}$ is [30, 65]. Details of the 11 constraints formulation can be found in  Yang and Hossein Gandomi (2012) and Koziel and Yang (2011).

$$\begin{aligned} &  f(x) = \sum ^{5}_{i=1} x_{i} x_{i+5} l_{i}, \\ &  g_{1}(x) = \frac{600P}{x_{5}x^{2}_{10}} - 14,000 \le 0, \\ &  g_{2}(x) = \frac{6P(l_{1}+l_{2})}{x_{4} x^{2}_{9}} - 14,000 \le 0, \\ &  g_{3}(x) = \frac{6P(l_{1}+l_{2}+l_{3})}{x_{3} x^{2}_{8}} - 14,000 \le 0, \\ &  g_{4}(x) = \frac{6P(l_{1}+l_{2}+l_{3}+l_{4})}{x_{2} x^{2}_{7}} - 14,000 \le 0, \\ &  g_{5}(x) = \frac{6P(l_{1}+l_{2}+l_{3}+l_{4}+l_{5})}{x_{1} x^{2}_{6}} - 14,000 \le 0, \\ &  g_{6}(x) = \frac{PL^{3}}{3E} \left( \frac{1}{l_{5}} + \frac{7}{l_{4}} + \frac{19}{l_{3}} + \frac{37}{l_{2}} + \frac{61}{l_{1}} \right) - 2.7 \le 0, \\ &  g_{7}(x) = \frac{x_{10}}{x_{5}} -20 \le 0, \\ &  g_{8}(x) = \frac{x_{9}}{x_{4}} -20 \le 0, \\ &  g_{9}(x) = \frac{x_{8}}{x_{3}} -20 \le 0, \\ &  g_{10}(x) = \frac{x_{7}}{x_{2}} -20 \le 0, \\ &  g_{11}(x) = \frac{x_{6}}{x_{1}} -20 \le 0. \end{aligned}$$

### Car side impact design

The goal of this test case is to minimize the car weight while meeting the compatibility requirements for the car crash test. Eleven variables are used to describe the requirements imposed on the design, materials, and reinforcement of B-Pillar, floor side, cross members, door beam, door beltline, roof rail, barrier height, and impact position during the side impact test. This problem is originally proposed by Gu et al. (2001) and the formulation is taken from Gandomi et al. (2011). The domain for this test case is $x_{1, 3\sim 7} \in [0.5, 1.5]$, $x_{2}\in [0.45, 1.35]$, $x_{8\sim 9} \in [0.192, 0.345]$, and $x_{10\sim 11}\in [-20, 0]$.

$$\begin{aligned} &  f(x) =1.98 + 4.90x_{1} + 6.67x_{2} + 6.98x_{3} \\  &  \quad + 4.01x_{4} + 1.78x_{5} + 2.73x_{7}, \\ &  g_{1}(x) = 1.16 - 0.3717x_{2}x_{4} - 0.00931x_{2}x_{10} \\  &  \quad -0.484x_{3}x_{9} + 0.01343x_{6}x_{10} -1 \le 0, \\ &  g_{2}(x) = 0.261 - 0.0159x_{1}x_{2} - 0.188x_{1}x_{8} \\  &  \quad - 0.019x_{2}x_{7} + 0.0144x_{3}x_{5} + 0.0008757x_{5}x_{10} \\ &  \quad + 0.08045x_{6}x_{9} + 0.00139x_{8}x_{11} + 0.00001575x_{10}x_{11} \\ &  \quad -0.9 \le 0, \\ &  g_{3}(x) = 0.214 + 0.00817x_{5} - 0.131x_{1}x_{8} - 0.0704x_{1}x_{9} \\  &  \quad + 0.03099x_{2}x_{6} -0.018x_{2}x_{7} + 0.0208x_{3}x_{8} + 0.121x_{3}x_{9} \\  &  \quad - 0.00364x_{5}x_{6} +0.0007715x_{5}x_{10} - 0.0005354x_{6}x_{10} \\  &  \quad +0.00121x_{8}x_{11} -0.9 \le 0, \\ &  g_{4}(x) = 0.74 -0.061x_{2} -0.163x_{3}x_{8} +0.001232x_{3}x_{10} \\  &  \quad -0.166x_{7}x_{9} +0.227x_{2}x_{2} -0.9 \le 0, \\ &  g_{5}(x) = 28.98 +3.818x_{3}-4.2x_{1}x_{2}+0.0207x_{5}x_{10} \\  &  \quad + 6.63x_{6}x_{9}-7.7x_{7}x_{8}+0.32x_{9}x_{10} -32\le 0, \\ &  g_{6}(x) = 33.86 +2.95x_{3}+0.1792x_{10}-5.057x_{1}x_{2} \\  &  \quad -11.0x_{2}x_{8}-0.0215x_{5}x_{10}-9.98x_{7}x_{8}+22.0x_{8}x_{9} \\  &  \quad -32 \le 0, \\ &  g_{7}(x) = 46.36 -9.9x_{2}-12.9x_{1}x_{8}+0.1107x_{3}x_{10} \\  &  \quad -32\le 0, \\ &  g_{8}(x) = 4.72 -0.5x_{4}-0.19x_{2}x_{3} \\  &  \quad -0.0122x_{4}x_{10} +0.009325x_{6}x_{10}+0.000191x_{11}^{2} \\ &  \quad -4\le 0, \\ &  g_{9}(x) = 10.58 -0.674x_{1}x_{2}-1.95x_{2}x_{8} \\  &  \quad +0.02054x_{3}x_{10} -0.0198x_{4}x_{10}+0.028x_{6}x_{10} \\  &  \quad -9.9\le 0, \\ &  g_{10}(x) = 16.45 -0.489x_{3}x_{7}-0.843x_{5}x_{6} \\  &  \quad +0.0432x_{9}x_{10} -0.0556x_{9}x_{11}-0.000786x_{11}^{2} \\  &  \quad -15.7 \le 0. \end{aligned}$$

### Keane bump

The Keane Bump benchmark is a scalable optimization test case proposed by Keane (1994) and it’s known to be challenging for GP-based BO methods. The goal is to perform minimization over the domain $[0,10]^{d}$. Here we use $d=18$ since this is the maximum dimension that can be passed into the model used for PFNs4BO (Müller et al. 2023).

$$\begin{aligned} f(x)= &  -\left| \frac{ \sum ^{d}_{i=1} \cos ^{4}(x_{i}) -2\prod _{i=1}^{d} \cos ^{2}(x_{i})}{\sqrt{\sum ^{d}_{i=1} ix_{i}^{2}}} \right| , \\ g_{1}(x)= &  0.75-\prod _{i=1}^{d} x_{i} \le 0, \\ g_{2}(x)= &  \sum _{i=1}^{d} x_{i} - 7.5d \le 0. \end{aligned}$$

## Footnotes

1 https://github.com/automl/PFNs4BO/.

## Declarations

### Replication of results

This paper provides detailed descriptions of the benchmark example in the “Appendix” to support result replication. The Python code for the benchmarks and GP- and PFN-based BO can be found in this GitHub repo https://github.com/rosenyu304/BOEngineeringBenchmark with tutorials for the use of each algorithms provided. The GPs are built with the BoTorch library, and the PFN model is taken from Müller et al. (2023).
